# The Anti-interferon Activity of Conserved Viral dUTPase ORF54 is Essential for an Effective MHV-68 Infection

**DOI:** 10.1371/journal.ppat.1002292

**Published:** 2011-10-06

**Authors:** Ronika Sitapara Leang, Ting-Ting Wu, Seungmin Hwang, Lidia T. Liang, Leming Tong, Jennifer T. Truong, Ren Sun

**Affiliations:** 1 Molecular Biology Institute, University of California Los Angeles, Los Angeles, California, United States of America; 2 Department of Molecular and Medical Pharmacology, University of California Los Angeles, Los Angeles, California, United States of America; University of California Berkeley, United States of America

## Abstract

Gammaherpesviruses such as KSHV and EBV establish lifelong persistent infections through latency in lymphocytes. These viruses have evolved several strategies to counteract the various components of the innate and adaptive immune systems. We conducted an unbiased screen using the genetically and biologically related virus, MHV-68, to find viral ORFs involved in the inhibition of type I interferon signaling and identified a conserved viral dUTPase, ORF54. Here we define the contribution of ORF54 in type I interferon inhibition by ectopic expression and through the use of genetically modified MHV-68. ORF54 and an ORF54 lacking dUTPase enzymatic activity efficiently inhibit type I interferon signaling by inducing the degradation of the type I interferon receptor protein IFNAR1. Subsequently, we show *in vitro* that the lack of ORF54 causes a reduction in lytic replication in the presence of type I interferon signaling. Investigation of the physiological consequence of IFNAR1 degradation and importance of ORF54 during MHV-68 *in vivo* infection demonstrates that ORF54 has an even greater impact on persistent infection than on lytic replication. MHV-68 lacking ORF54 expression is unable to efficiently establish latent infection in lymphocytes, although it replicates relatively normally in lung tissues. However, infection of IFNAR−/− mice alleviates this phenotype, emphasizing the specific role of ORF54 in type I interferon inhibition. Infection of mice and cells by a recombinant MHV-68 virus harboring a site specific mutation in ORF54 rendering the dUTPase inactive demonstrates that dUTPase enzymatic activity is not required for anti-interferon function of ORF54. Moreover, we find that dUTPase activity is dispensable at all stages of MHV-68 infection analyzed. Overall, our data suggest that ORF54 has evolved anti-interferon activity in addition to its dUTPase enzymatic activity, and that it is actually the anti-interferon role that renders ORF54 critical for establishing an effective persistent infection of MHV-68.

## Author Summary

Human gammaherpesviruses, Kaposi's sarcoma-associated herpesvirus and Epstein-Barr virus, are the cause of several malignancies, especially in patients immunocompromised due to HIV infection. The study of these human gammaherpesviruses is difficult due to their inability to replicate in cell culture and the lack of a small-animal model. Murine gammaherpesvirus-68 is a genetically and biologically similar virus that is utilized as a mouse model because it offers such advantages as the ability to replicate in cell culture, a manipulatable genome, and infection of mice. In this study, we have identified viral open reading frame 54 (ORF54) as an inhibitor of innate immunity, specifically of the type I interferon response. Although ORF54 is a conserved viral dUTPase, we found that its anti-interferon activity does not require its enzymatic activity. Through infection of cells and mice, we define the critical role of ORF54 in establishing persistent latent infection of MHV-68 by inducing the degradation of the type I interferon receptor. Our studies provide new insights into the far reaching effects of type I interferon signaling and the dual role of ORF54. This work could aid in the development of vaccine strategies to gammaherpesvirus infection.

## Introduction

Virus infection induces numerous immune responses in the host, the earliest of which is the innate immune response [1], [2]. The innate immune response is comprised of many layers of non-specific defense, including anatomical barriers, such as skin and mucosa, the complement system, inflammation, and various cells, such as natural killer cells, phagocytes, mast cells, macrophages, dendritic cells, neutrophils, and basophils [3]–[5]. The innate immune response plays a crucial role in shaping the ensuing adaptive immune response, in part by the production of cytokines in response to infection [2], [6]. Interferons (IFN) are cytokines secreted upon virus infection that induce the expression of a variety of antiviral gene products, reducing virus replication and further infection [1], [7]–[9].

Interferons are classified as type I and II, as defined by the cell types able to produce them and the receptors they bind to [1]. Unlike the type II IFN-γ that is produced by specific cells of the immune system, IFN-α and IFN-β are type I IFNs that can be produced in most cell types [10]. Mammals encode a single IFN-β and several IFN-α species. All type I IFN species bind to the same ubiquitously expressed receptor, called the type I interferon receptor, or IFNAR [11]. This receptor is a heterodimer comprised of IFNAR1 and IFNAR2 [12]. Although normally unassociated, IFNAR1 and IFNAR2 dimerize upon the binding of IFN-α or IFN-β first to IFNAR2, and then to both receptors in the dimer [13]. IFNAR1 and IFNAR2 are each pre-associated with one of the members of the Janus protein tyrosine kinase family, where TYK2 is associated with IFNAR1 and JAK1 with IFNAR2. IFN binding and formation of the complete IFNAR dimer leads to cross-phosphorylation of TYK2 and JAK1, and the phosphorylation of the IFNAR chains they are permanently associated with. These phosphorylation events set up a platform for the recruitment of STAT1 and STAT2, which in turn are also phosphorylated. The phosphorylated STAT proteins dimerize prior to joining with IFN-regulatory factor 9 (IRF9) to form the Interferon-Stimulated Gene Factor 3 (ISGF3γ) transcription factor, which translocates to the nucleus where it induces the expression of hundreds of interferon stimulatory genes (ISG) (reviewed in [1]).

Herpesviruses are large, double-stranded DNA viruses defined by their ability to persist for the lifetime of the host by establishing latent infections and by evading the host immune response [14]. Both lytic and latent infections of herpesviruses are able to directly cause disease [15]–[23]. Gaining understanding of the mechanisms by which herpesviruses maintain persistent infections and evade immune surveillance is a key step in controlling the diseases they are associated with. Herpesviruses are subdivided into alpha, beta, and gamma herpesviruses [14]. The human gammaherpesviruses are Epstein-Barr virus (EBV) and Kaposi's sarcoma-associated herpesvirus (KSHV). Both EBV and KSHV are associated with malignancies; EBV with Burkitt's lymphoma, nasopharyngeal carcinoma, and oral hairy leukoplakia and KSHV with Kaposi's sarcoma, primary effusion lymphoma, and multicentric Castleman's disease [23]–[28]. We use the biologically and genetically related virus, murine gammaherpesvirus-68 (MHV-68) as a model to study the human gammaherpesviruses [29]–[32]. Like KSHV, MHV-68 is a gamma-2-herpesvirus. MHV-68 establishes lytic and latent infections in mice [33], replicates readily in *in vitro* cell culture systems, and has a genome that can be genetically manipulated by utilizing a bacterial artificial chromosome (BAC) system [34].

In KSHV and MHV-68 several studies have identified viral proteins involved in the inhibition of the host innate and adaptive immune responses. In particular, KSHV open reading frame (ORF) 10 binds to JAK and STAT proteins to block IFN mediated signaling [35]. KSHV and MHV-68 ORF36 bind to phosphorylated IRF3, thus inhibiting the production of IFN-β [36]. KSHV ORF45 interacts with and inhibits IRF7 from entering the cell nucleus [37]. The KSHV immediate early transcription factor and E3 ligase, RTA, targets IRF7 for protein degradation [38]. Other viral ORFs also contribute to immune evasion by inducing the downregulation of surface molecules critical for immune activation. K5 of KSHV directs the downregulation of Tetherin/BST2, ICAM, and MHC class I [39]–[42]. Furthermore, K3 of both KSHV and MHV-68 inhibit the surface expression of MHC class I [39], [43].

2′-deoxyuridine 5′-triphosphate pyrophosphatase (dUTPase) reduces the misincorporation of uracil in the DNA genome by controlling the level of dUTP through conversion of dUTP into dUMP, ultimately leading to an increased amount of dTTP and a lower dUTP∶dTTP ratio [44]. This enzyme can be found in all classes of organisms and in many RNA and DNA viruses as well [45], [46]. In this study, we define the role of viral ORF54, a functional dUTPase, in evading the host innate immune response to virus infection. In an effort to understand these two separate functions of ORF54, we analyzed the signaling pathways altered by wild-type ORF54 and a dUTPase-null mutant. Through *in vitro* and *in vivo* infection, in wild-type and transgenic mice, with recombinant MHV-68 harboring mutations in ORF54, we found that ORF54 interferes with-type I interferon signaling, which further affects persistent infection and the establishment of latency.

## Results

### Identification of ORF54 as an inhibitor of the type I interferon response

**Figure 1:**
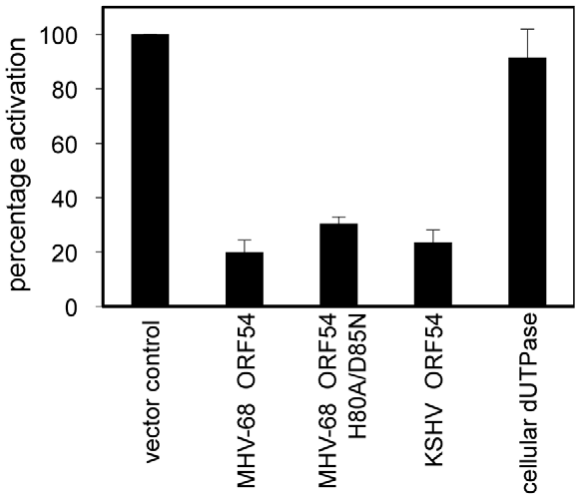
MHV-68 and KSHV ORF54 diminish IFN-α induced activation of ISRE in a non-dUTPase related manner. HEK 293T cells were transiently transfected with ISRE_firefly-luciferase, PGK_renilla-luciferase as an internal control, and with expression plasmids for each ORF, control plasmid, or murine cellular dUTPase, then were treated with 3×10^4^ U/mL of human IFN-α. Firefly luciferase values were normalized to renilla luciferase. Percentage of activation is calculated in comparison to control, an unrelated MHV-68 ORF or vector control. Error bars depict one standard error based on four independent trials.

To systematically identify MHV-68 viral ORFs that inhibit type I IFN signaling, we conducted a screen where 293T cells were transiently transfected with a reporter construct containing firefly luciferase driven by the interferon-stimulated response element (ISRE_firefly-luciferase). Cells were also co-transfected with a reporter construct containing renilla luciferase driven by the constitutively active PGK promoter and each MHV-68 viral ORF or a vector control. Transfected cells were treated with human IFN-α and the induction of the ISRE reporter was measured by dual luciferase assay. Since this screen examines cellular responses after IFN-α treatment, the viral proteins previously identified to inhibit IRF3 or IRF7 signaling, thus preventing the induction of type I IFN production, would not necessarily be identified. Of all the MHV-68 viral ORFs that were screened, we found 8 ORFs that were potentially able to inhibit type I IFN signaling to a level that is 50% of the activation seen with vector control. Two of them are M2 and M8, ORFs specific to MHV-68. M2 has been previously shown to inhibit IFN signaling [47], thus validating our screen. Among the other 6 ORFs that also have homologues in KSHV and EBV, ORFs 10, 11, and 54 are particularly interesting because a previous sequence analysis study identified a shared dUTPase-related domain, although only ORF54 contains catalytic active sites [48]. Since ORF54 is a viral dUTPase and is one of the top three strongest inhibitors, our following study focuses on its potential anti-IFN function and the biological significance of this function during viral infection. Cells transfected with ORF54 demonstrated a diminished activation of ISRE following treatment with IFN-α (Figure 1), with only 20% of the activation seen in control transfections. KSHV ORF54 also demonstrated diminished activation of ISRE, at 23% of control (Figure 1), suggesting that the ability to inhibit type I IFN signaling is a gammaherpesvirus conserved function of ORF54.

### ORF54 inhibition of the type I interferon response is independent of enzymatic activity

**Figure 2:**
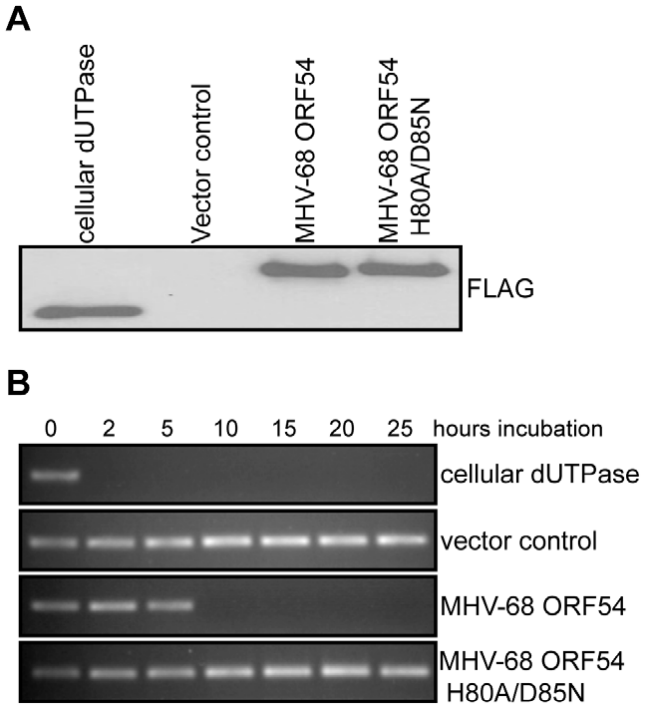
Generation of a dUTPase-null MHV-68 ORF54. **A**) Immunoblot against the FLAG epitope of immunoprecipitated ORFs or control demonstrates equal amounts of each protein was purified from transfected 293T cells and used in the dUTPase activity assay. **B**) A PCR reaction utilizing individual dNTPs, with dUTP replacing dTTP, was used to determine the presence of functional dUTP after incubation (between 0 and 25 hours) with purified murine cellular dUTPase, MHV-68 ORF 54, MHV-68 ORF54 H80A/D85N, and a vector control. The presence of a PCR product indicates intact dUTP, and thus a non-functional dUTPase.

To test whether the dUTPase function is required for the anti-IFN activity of ORF54, we constructed a catalytic domain mutant of MHV-68 ORF54 by replacing the amino acid histidine at position 80 with alanine and the amino acid aspartic acid at position 85 with asparagine (ORF54 H80A/D85N). These two amino acids were chosen for mutagenesis due to their proximal location in the putative active site of ORF54 dUTPase and their predicted importance for enzymatic reaction. Viral and non-viral dUTPases typically share five highly conserved motifs, although the arrangement found in herpesviruses is different compared to human dUTPase [44], [48]. Several studies have identified the presence of dUTPase motif III, which is critical for catalytic activity, in the herpesvirus dUTPases [44]. Aspartic acid at position 85, located in motif III, was altered because aspartic acids at positions 84 and 86 in human endogenous retrovirus (HERV-K) were found to be critical for catalytic activity, but not for dUTP binding [44]. Histidine at position 80 was chosen because it is conserved in gammaherpesviruses, and in EBV a histidine at position 71 contains a necessary imidazole group [49]. The ORF54 H80A/D85N mutant demonstrates a complete loss in enzymatic activity, although the protein expression level remained the same (Figure 2). When co-transfected with ISRE_firefly-luciferase, ORF54 H80A/D85N maintains the ability to diminish IFN-α induced activation of the ISRE to 32% of the activation seen in control samples (Figure 1). This result suggests ORF54 inhibition of the type I IFN signaling cascade is independent of its dUTPase enzymatic activity. To further clarify if the dUTPase function was sufficient to inhibit type I IFN signaling, we tested the ability of murine cellular dUTPase to inhibit IFN-α induced activation of ISRE in our reporter assay. The cellular dUTPase was unable to block activation of ISRE_firefly-luciferase (Figure 1), further indicating that dUTPase enzymatic activity does not necessarily correlate with anti-IFN activity.

### ORF54 induces the degradation of the type I interferon receptor 1

**Figure 3:**
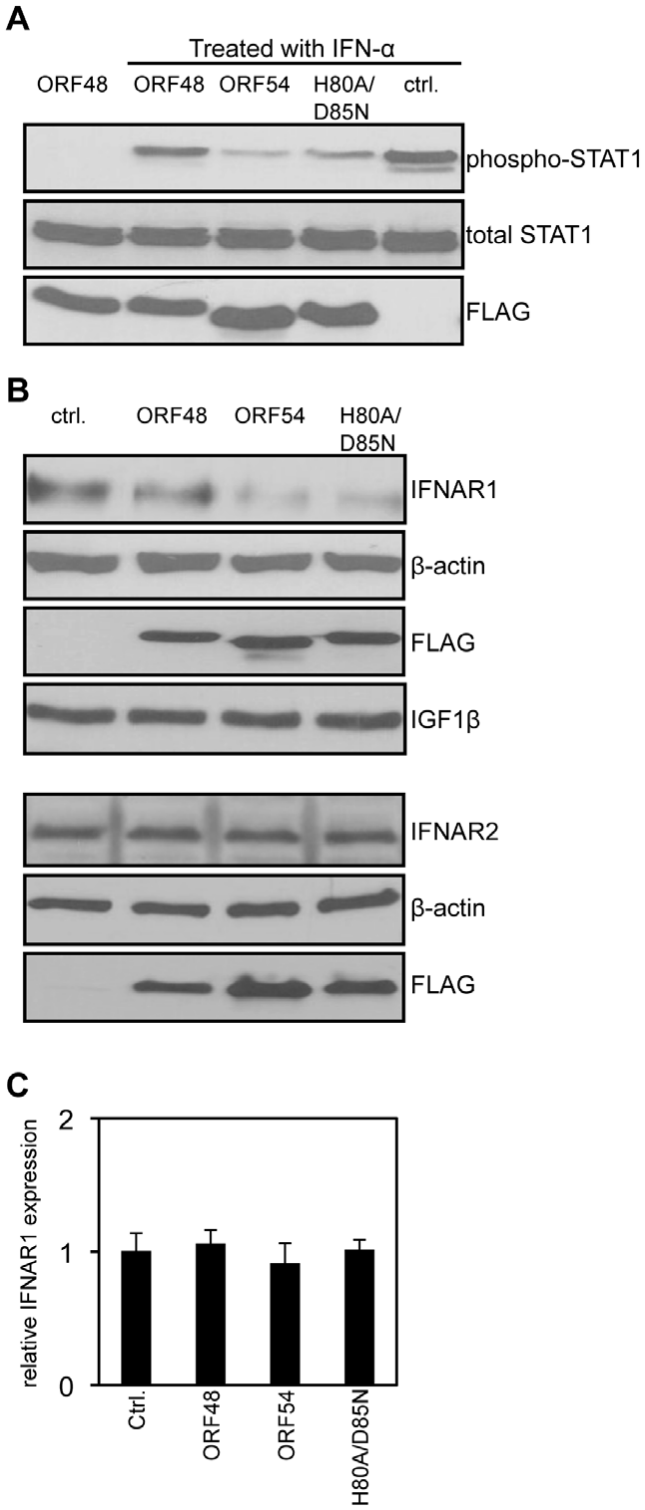
ORF54 induces degradation of IFNAR1. 24 hours post transfection, 293T cells were treated with 1 µg/mL of puromycin to select for transfected cells. 48 hours post treatment, cells overexpressing vector control or FLAG-tagged MHV-68 ORFs were: **A**) treated with human IFN-α for 15 minutes. Immunoblot for phosphorylation of STAT1 on Tyr 701 was assayed first; the membrane was subsequently stripped twice to demonstrate equal levels of total STAT1 and expression of FLAG-ORFs as controls; **B**) Upper panels: Lysed to analyze level of IFNAR1. The membrane was subsequently stripped to demonstrate equal expression of the FLAG-ORFs and β-actin as controls, and IGF1β to demonstrate specificity of ORF54 to IFNAR1. Lower panels: The same lysates were also subjected to immunoblot against IFNAR2. The membrane was stripped twice to demonstrate equal expression of FLAG-ORFs and β-actin as controls. **C**) harvested for total RNA isolation. Human IFNAR1 transcript levels were quantified by RT-PCR, normalized first to actin, and are shown relative to vector control transfected cells. Ctrl. is the vector control, ORF48 is an unrelated MHV-68 ORF used as a negative control.

The type I IFN signaling pathway begins with the binding of IFN-α to the surface IFNAR and results in the production of ISGs [7], [8]. As each step in the JAK/STAT pathway that ensues is well defined [1], we aimed to identify the step where ORF54 exerts its function. We first assayed a central event in type I IFN signaling, the phosphorylation of STAT1 protein. 293T cells ectopically overexpressing MHV-68 ORF54 or ORF54 H80A/D85N both demonstrated a reduced level of phosphorylation of STAT1 following treatment by IFN-α in comparison to cells transfected with vector control or an unrelated MHV-68 ORF (Figure 3A). By assay of steps upstream of the phosphorylation of STAT1, we show that cells expressing MHV-68 ORF54 or ORF54 H80A/D85N both demonstrated a reduced level of total IFNAR1 (Figure 3B). As a control for specificity, we also found that ORF54 and ORF54 H80A/D85N do not alter levels of the surface protein type I insulin-like growth factor receptor-β (IGF1β) or IFNAR2 (Figure 3B). These results suggest that ORF54 induces the degradation of IFNAR1 independently of its dUTPase enzyme activity, and that this degradation results in a reduction of the type I IFN response, including the phosphorylation of STAT1. The transcript level of IFNAR1 remains comparable between the vector control and viral ORF transfected cells (Figure 3C), suggesting that the ORF54 induced reduction of IFNAR1 is at the protein level.

### ORF54 plays a role in counteracting IFN during viral replication

**Figure 4:**
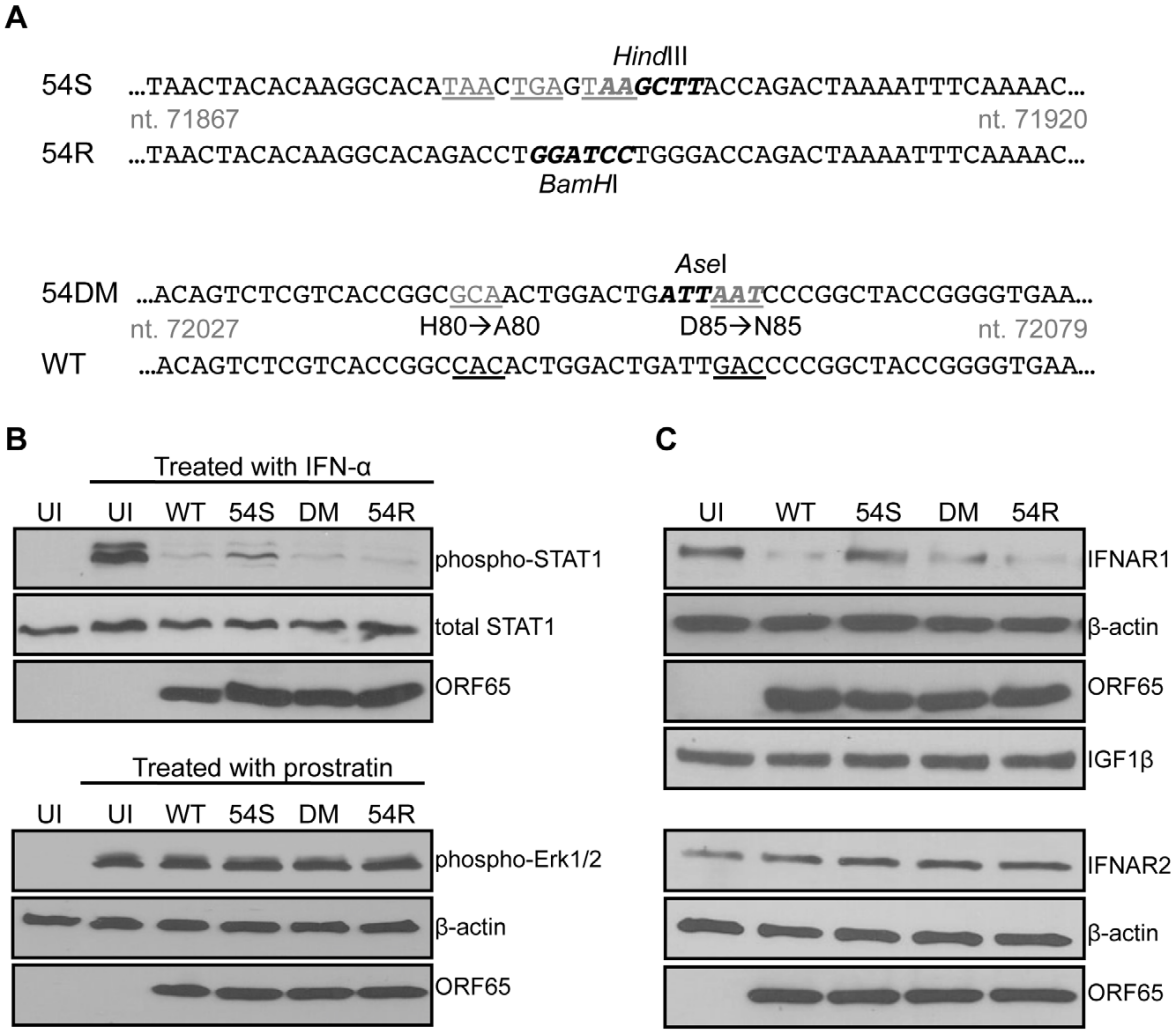
An ORF54-null virus has incomplete degradation of IFNAR1. **A**) Schematic diagram depicting the construction of an MHV-68 ORF54 mutant with an insertion of a triple translational stop codon (54Stop), its revertant (54R), and a dUTPase-null double amino acid mutant with ORF54 histidine at position 80 mutated to alanine and aspartic acid at position 85 mutated to asparagine (54DM). Nucleotide coordinates are based on GenBank U97553. WT MHV-68 and 54R contain a *BamH*I restriction enzyme site that is altered to a *Hind*III site with the introduction of the triple stop codon. The base pair changes in 54DM allow the introduction of an *Ase*I site that is absent in WT MHV-68. Base changes are depicted in gray and underlined; restriction enzyme recognition sites are bolded. **B**) Upper panels: NIH3T3 cells infected with each indicated virus at MOI 2 for 24 hours were treated with murine IFN-α for 15 minutes and then harvested for immunoblot against phosphorylation of Tyr701 of STAT1. Blots were stripped twice to demonstrate equal amounts of total STAT1 and comparable infection with expression of viral protein ORF65. Lower panels: NIH3T3 cells infected with each indicated virus at MOI 2 for 24 hours were treated with a final concentration of 160 µM of prostratin for 30 minutes and then harvested for immunoblot against phosphorylation of Thr202 and Tyr204 of Erk1/2. Blots were stripped twice to demonstrate equal amounts of β-actin and comparable infection with expression of viral protein ORF65. **C**) 293T cells infected with the indicated viruses at MOI 1 for 24 hours were lysed and analyzed for total amount of IFNAR1. Blots were stripped to demonstrate equal loading with β-actin, comparable infection with expression of viral protein ORF65, and specificity of ORF54 to IFNAR1 with IGF1β. The same lysates were also subjected to immunoblot against IFNAR2. The membrane was stripped twice to demonstrate equal loading with β-actin and comparable infection with expression of viral protein ORF65.UI = uninfected; 54S = 54Stop; DM = 54DM.

We generated three recombinant MHV-68 to study the importance of ORF54 (Figure 4A). Because ORF54 is not required for the virus to replicate in cultured cells [50], the first mutant is an ORF54-null virus that has triple translational stop codons inserted near the N-terminus of MHV-68 ORF54 (54Stop). The second virus is a revertant for 54Stop, where the translational stop codons have been removed and reverted back to wild type (54R). This virus ensures that any phenotype demonstrated with 54Stop is due to the ORF54-null mutation and not any additional recombination or unintentional mutagenesis of the MHV-68 viral genome. The third virus (54DM) has the same two amino acid mutations that abolish dUTPase activity as in the ORF54 H80A/D85N expression construct (Figure 4A).

NIH3T3 cells were infected with an MOI of 2 to initiate single-step growth kinetics. 24 hours post infection, cells were either treated for 15 minutes with 500 units/mL of mouse IFN-α or left untreated. Uninfected cells were also treated as a control. Immunoblots probing for the phosphorylation of STAT1 protein demonstrate that while wild-type (WT) MHV-68, 54DM, and 54R are able to block STAT1 phosphorylation, higher levels of phosphorylated STAT1 are found in cells infected with 54Stop (Figure 4B). Total STAT1 is comparable in all samples and all cells are infected to a similar level, as demonstrated by equal production of the MHV-68 late protein ORF65. Therefore, this result supports the role of ORF54 in the inhibition of type I IFN signaling during viral infection, as lacking ORF54 partially alleviates the block. However, we also noted that the phosphorylated STAT1 seen in cells infected with 54Stop is still not at the level observed in uninfected and treated cells. This is perhaps due to the other viral ORFs that are present and still able to inhibit the type I IFN signaling pathway, as evidenced by the multiple ORFs identified in our original screen. As an additional control to demonstrate that infected cells are still capable of responding to stimuli, NIH3T3 cells were identically infected with an MOI of 2 and treated at 24 hours post infection with 160 µM prostratin (12-deoxyphorbol 13-acetate) for 30 minutes (Figure 4B). Prostratin initiates a signaling cascade that induces the reactivation of latent viruses [51]. In this control assay, immunoblots probing for the phosphorylation of p44/p42 (Erk1/2) demonstrate that infected cells are viable enough to respond to stimuli even 24 hours post infection. These studies demonstrate that although at this MOI the 54Stop virus can establish a robust viral infection, the lack of functional ORF54 makes it unable to effectively block the resulting induction of the type I IFN signaling cascade.

Since we showed the degradation of IFNAR1 in the presence of overexpressed ORF54 (Figure 3B), we assayed this phenotype during virus infection to determine the biological relevance. By infecting cells with WT MHV-68 and 54R we found that IFNAR1 is degraded (Figure 4C). This degradation is reduced in cells infected with 54Stop virus, suggesting that ORF54 is required for the virus to induce degradation of IFNAR1. Furthermore, because cells infected with 54DM virus show a similar level of IFNAR1 reduction as with WT or 54R infection, this indicates that ORF54 dUTPase enzymatic function is not required for the degradation of IFNAR1 and the inhibition of the type I IFN signaling cascade. Interestingly, unlike with STAT1 phosphorylation, we observed a comparable level of IFNAR1 between 54Stop-infected cells and uninfected cells, indicating that ORF54 is the sole viral protein responsible for IFNAR1 degradation during viral infection. As a control for specificity, we also found that virus infection does not alter the levels of IFNAR2 or the surface protein IGF1-β (Figure 4C). Therefore, ORF54 mediated degradation is not a general phenomenon for all surface proteins and IFNAR1 is a specific target for such ORF54 function. The transcript level of IFNAR1 remains comparable amongst cells infected with WT, 54Stop, 54DM, and 54R viruses (Figure S1), suggesting that ORF54 does not alter the transcription of IFNAR1 and the ORF54 induced degradation of IFNAR1 is at the protein level.

**Figure 5:**
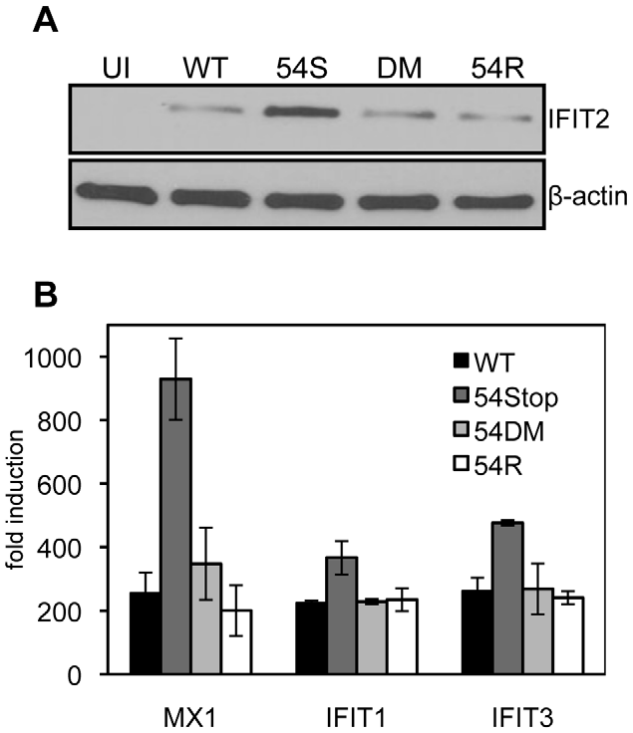
An ORF54-null virus infection promotes higher induction of interferon stimulated genes. Bone marrow derived macrophages from wild type mice were infected at an MOI of 2. Equal infection was ensured by RT-PCR quantifying input viral genome copies from total infected cellular DNA 1 hour after virus was introduced to the culture. **A**) 24 hours post infection cells were harvested for immunoblot against murine IFIT2. The blot was stripped to demonstrate equal expression β-actin as a control. UI = uninfected; 54S = 54Stop; DM = 54DM. **B**) 24 hours post infection, RNA was harvested and reverse transcribed to cDNA. Transcripts for murine MX1, IFIT1, and IFIT3 were measured by real-time PCR. The fold induction was calculated by first normalizing each C(t) for the transcript to the actin C(t), and then comparing to uninfected samples.

Because we found 54Stop defective in inhibiting type I IFN signaling due to its inability to induce IFNAR1 degradation, we assayed the downstream induction of ISGs following infection of bone marrow derived macrophages. Macrophages were chosen for infection due to their high endogenous induction of anti-viral genes following virus infection. 24 hours post infection at an MOI of 2, cells were harvested for immunoblot against the ISG IFIT2. All infected cells show induced expression of IFIT2 compared to uninfected cells. IFIT2 protein expression was highest in 54Stop infected cells compared to in cells infected with WT, 54DM, and 54R (Figure 5A), suggesting a virus lacking functional ORF54 is not as effective in blocking ISG induction as WT MHV-68. Additionally, total RNA was harvested from infected macrophages to measure the transcript level of several ISGs, including MX1, IFIT1, and IFIT3. In all cases, the ISG induction in cells infected with 54Stop is higher than with infection by WT, 54DM, and 54R, suggesting ORF54 has a role in inhibition of type I IFN responses (Figure 5B).

### ORF54 is required for efficient growth in the presence of type I IFN signaling

**Figure 6:**
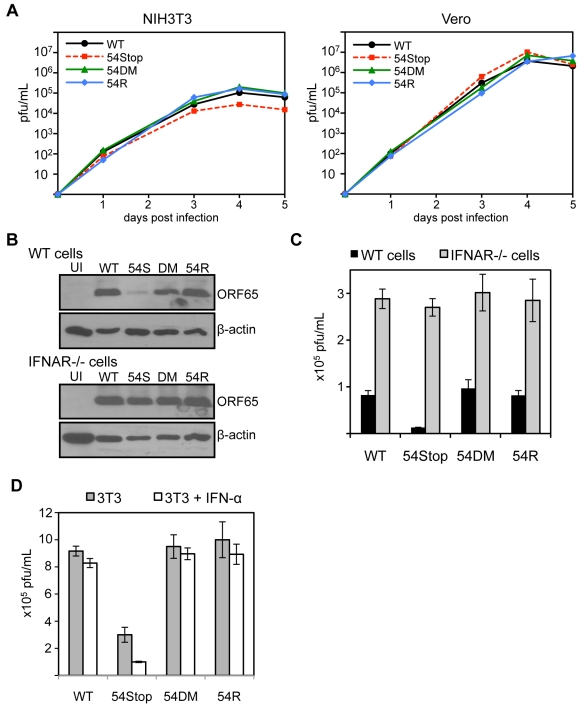
ORF54-null virus has a moderate defect in the presence of type I IFN signaling. **A**) In these multiple-step growth curves, NIH3T3 or Vero cells were infected at MOI 0.01 and harvested for 5 consecutive days post infection. Viral titers were calculated by plaque assay following three freeze and thaw cycles of the whole cell and supernatant lysate. This data is one representative from 3 independent trials. NIH3T3 curves are shown in the left panel, Vero curves in the right. **B**) Bone marrow derived macrophages isolated from IFNα/β receptor knock-out mice and wild-type controls were infected with each indicated virus at MOI 4. 60 hpi, cells were harvested and immunoblotted for viral late antigen ORF65. The blots were stripped and re-probed for β-actin. UI = uninfected; 54S = 54Stop; DM = 54DM. **C**) Infection was completed as described in B. At 60 hpi, cells were harvested and viral titers were calculated by plaque assay following three freeze and thaw cycles of the whole cell and supernatant lysate. The assay was done in triplicate; averages are shown here with error bars reflecting one standard error. For 54Stop compared to WT, 54DM, and 54R, p-values are 0.0004, 0.003, 0.0007, respectively. **D**) NIH3T3 cells were left alone or pretreated with 100 units/mL of IFN-α one day prior to infection by each indicated virus at MOI 0.01. Immediately following and 2 days post infection, IFN-α was supplemented into the media at 100 units/mL in the treated samples. On 4 days post infection, infected samples were harvested and subjected to three freeze and thaw cycles prior to titer by plaque assay. The assay was done in triplicate; averages are shown here with error bars reflecting one standard error. For 54Stop compared to WT, 54DM, and 54R, p-values are 0.0007, 0.003, 0.008, respectively. For 54Stop, the p-value comparing untreated and IFN-α treated cells is 0.022.

Although ORF54 is not essential for MHV-68 replication, the 54Stop virus exhibits a defect in STAT1 activation and downregulating IFNAR1 expression. Hence, we speculated that lack of ORF54 might have some effect on viral replication in cells that are capable of producing and responding to type I IFN, such as NIH3T3 cells. Indeed, as shown in Figure 6A, we found that the 54Stop virus has moderately attenuated multiple-step growth in NIH3T3 cells (approximately 3.8-fold on day 4 and 4.1-fold attenuation on day 5 post infection compared to WT). Moreover, this attenuation of the 54Stop virus was not observed in Vero cells (Figure 6A), which are unable to produce type I IFN in response to infection [52]. To ensure this difference in phenotype of the 54Stop virus is due to its inability to block IFN signaling, we infected bone marrow derived macrophages from wild-type and IFN α/β receptor knockout (IFNAR-/-) mice at an MOI of 4. By immunoblot of infected wild-type macrophages, we see a lower production of the MHV-68 capsid protein, ORF65, with 54Stop infection compared to WT, 54DM, and 54R infection. However, the ORF65 production with 54Stop infection is rescued in the IFNAR−/− macrophages (Figure 6B). Similarly, the infectious titer produced from wild-type macrophages infected with 54Stop is between 6.3- to 7.5-fold lower than that of WT, 54DM, and 54R, while the infectious titers produced from IFNAR−/− macrophages are similar amongst all four virus types (Figure 6C). To further demonstrate the role of ORF54 in antagonizing IFN signaling during viral replication, virus production in NIH3T3 cells with or without the treatment of 100 units/mL of IFN-α was compared (Figure 6D). The peak viral titers of WT, 54DM, and 54R viruses were relatively unaffected at this low dose, while the average drop for 54Stop was 3-fold, p-value = 0.022. The growth defects observed with 54Stop in the presence of type I IFN response demonstrate the role of ORF54 in antagonizing the signaling pathway. The consistent, but modest, defects seen with 54Stop infection are also meaningful as it is known the virus has multiple anti-IFN genes [34]–[38], [53], [54]. Additionally, all of above *in vitro* viral replication results demonstrate that 54DM behaved similarly to WT and 54R viruses but not to 54Stop, indicating that the anti-IFN function of ORF54 is independent of its dUTPase enzymatic function.

### ORF54 plays a significant anti-interferon role during the establishment of MHV-68 viral latency *in vivo*

**Figure 7:**
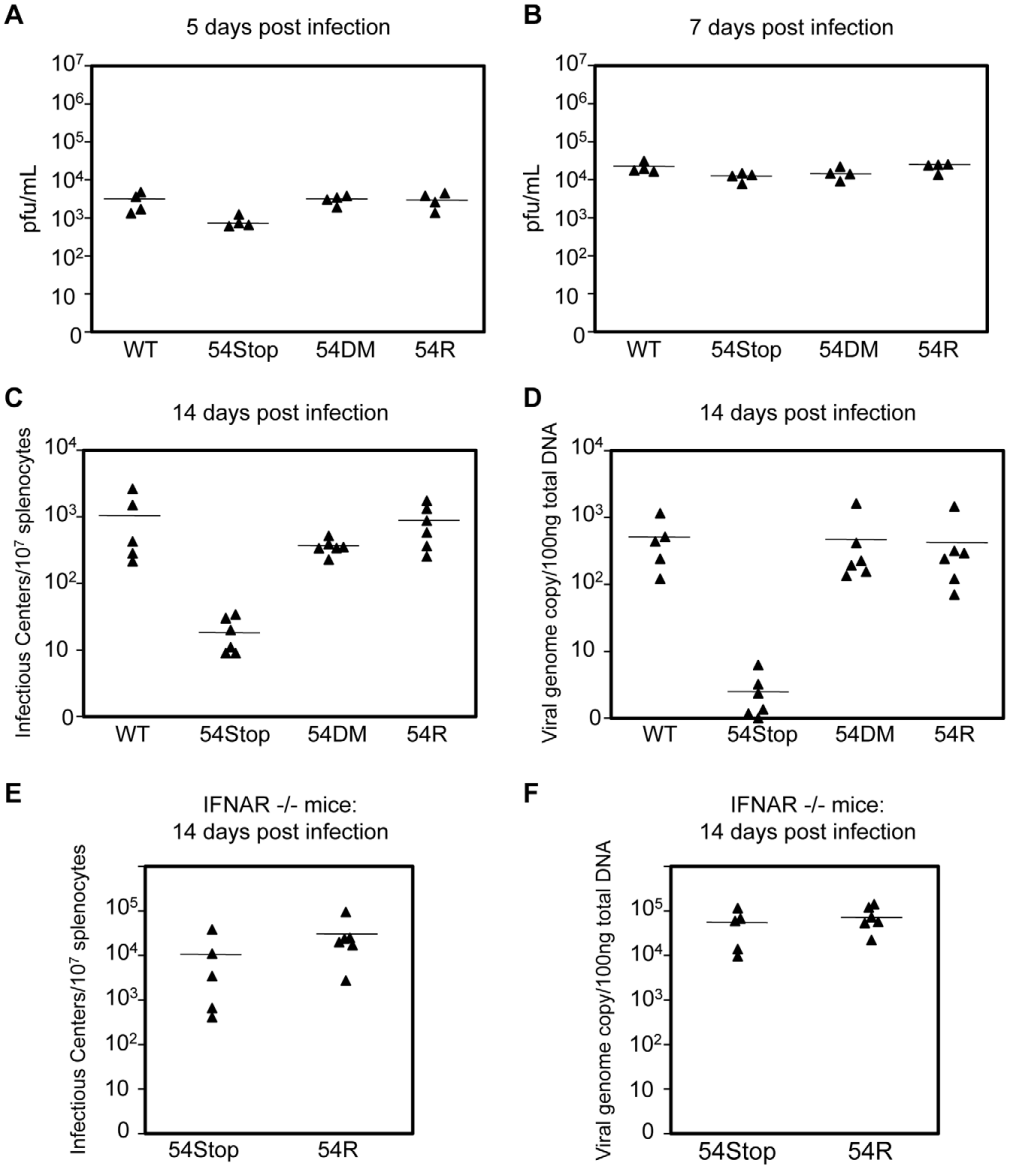
ORF54 non-dUTPase function is essential for establishment of latency *in vivo*, but not lytic infection. Balb/C mice were infected with 500 pfu of each indicated virus. (**A–B**) Lung tissue was harvested for plaque assay to measure infectious titer. Infectious titer from lung lysates at **A**) 5 dpi and **B**) 7 dpi. P-values for 54S compared to WT, 54DM, and 54R in A are 0.047, 0.002, 0.018, respectively and in B are 0.048, 0.396, 0.022, respectively. (**C–D**) At 14 dpi, splenocytes were harvested to **C**) measure infectious centers which are derived from reactivating virus and **D**) to determine viral genome copies from 100 ng of total splenocyte DNA. P-values for 54Stop compared to WT, 54DM, and 54R in C are 0.043, 0.0003, 0.005, respectively, and in D are 0.007, 0.042, and 0.040, respectively. P-value of 54DM compared to WT in C is not significant, p = 0.159. **E**) IFNAR−/− mice were infected with 500 pfu of 54Stop and 54R viruses. At 14 dpi, splenocytes were harvested to measure infectious centers, the p-value between 54Stop and 54R is not significant, p = 0.248, and **F**) viral genome copies determined from 100 ng of total splenocyte DNA, the p-value between 54Stop and 54R is not significant, p = 0.387. In all Figures, each ▴ represents one animal, and a bar marks averages. For A,B n = 4; for C,D n = 6 except for WT virus where n = 5; for E and F, n = 5 for 54Stop and n = 6 for 54R.

We examined the role of ORF54 *in vivo* by infecting Balb/C mice with WT MHV-68, 54Stop, 54DM, or 54R and measuring lytic replication in the lungs on 5 and 7 days post infection (dpi) and the establishment of latency at its peak on 14 dpi. Lytic infection does not critically require ORF54. With intranasal infection we found that lytic replication in the lungs at 5 dpi appears to be only slightly affected by the lack of ORF54. The average infectious titer was approximately 3.5- to 3.8-fold lower with 54Stop infection than with WT MHV-68, 54DM, or 54R, with p-values 0.047, 0.002, and 0.018, respectively (Figure 7A). At 7 dpi the effects from the lack of ORF54 on the lung viral titers were even smaller (1.2- to 1.8-fold lower than others), and possibly insignificant with p-values of 0.048 for WT, 0.396 for 54DM, and 0.022 for 54R (Figure 7B). Similar results and trends were obtained with analysis of the viral genome copy number in the lung lysates (Figure S2). Infection of 54DM was comparable to WT and 54R viruses, indicating that function(s) other than dUTPase activity of ORF54 play a role, however minor, during productive infection in the lung.

At 14 dpi, we analyzed the establishment of splenic latency by performing an infectious center assay and quantifying viral genome copy numbers of the spleens of mice infected with WT MHV-68, 54Stop, 54DM, or 54R. The infectious center assay quantifies the amount of latent virus that reactivates from a population of B-cells upon *ex vivo* culturing. We found that the 54Stop virus has approximately 53- and 45-fold less reactivated virus per 10^7^ lymphocytes compared to WT and 54R viruses, respectively (Figure 7C). Quantitation of the viral genome copies is an unbiased measurement that does not rely on virus activity in the assay itself, and it showed an even more pronounced reduction. The 54Stop genome is almost undetectable, with 140- to 164-fold lower genome copies than WT MHV-68, 54DM, and 54R (Figure 7D). Lower levels of viral genome copies and infectious centers suggest the defect of the 54Stop virus is in the establishment of latency and not in the ability to reactivate. If this drastic reduction in 54Stop latency is due to the inability of the virus to block type I IFN signaling, we would expect to see latency of 54Stop rescued in IFNAR−/− mice, which lack the type I IFN receptor. Indeed, in IFNAR−/− mice splenic latency of 54Stop at 14 dpi is greatly increased to a level similar to 54R virus; the number of infectious centers with 54Stop infection is 2.8-fold lower than with 54R, but with a statistically insignificant p-value of 0.248 (Figure 7E). Viral genome copies of 54Stop and 54R viruses from IFNAR−/− mice splenocytes are nearly identical (Figure 7F). Our results indicate that ORF54 is required for establishing a latent infection, and this requirement is based on its anti-IFN activity as the deficiency of 54Stop is rescued in mice unresponsive to type I interferon. This result concludes that the major role of ORF54 for establishing and/or maintaining latency is to inhibit type I interferon responses.

## Discussion

Viral pathogens have adapted several avenues of immune evasion, including inhibition of the innate immune response. The importance of the interferon system is highlighted by the multiple evasion strategies employed by viruses, such as herpesviruses [14], [55]. Here we present a report on ORF54, a conserved viral dUTPase, identified by our unbiased screen designed to isolate gammaherpesvirus ORFs involved in the inhibition of the type I IFN induced signaling pathway. Interestingly, ORF54 enzymatic activity proves dispensable for its anti-IFN function. Using KSHV ORF54 we were further able to demonstrate the anti-IFN role of ORF54 is conserved amongst KSHV and MHV-68. We have found that when high levels for ORF54 are present, either by ectopic expression or by infection with MHV-68, the total amount of cellular type I interferon receptor 1 is reduced, causing depression of the type I IFN response. Finally, through manipulation of the viral genome and murine host, we uncovered the biologically relevant function that ORF54 plays in not only blocking a critical component of the innate immune response, but also in persistent infection of gammaherpesviruses.

The most surprising and significant finding of our study is that the primary role of ORF54 during MHV-68 infection of cells and mice appears to be its anti-IFN function rather than its dUTPase activity. In three separate cell culture systems where type I IFN signaling is functioning, we demonstrate attenuation of the 54Stop virus, but not 54DM, a dUTPase-null virus (Figure 6). None of the defects seen with the 54Stop virus are observed with 54DM infection, indicating that the dUTPase activity is dispensable for the role of ORF54 during MHV-68 replication in cultured cells. In mice, while the 54Stop virus has a relatively normal productive infection in the lung, it shows a very strong deficit in spleen latency that is not found in the dUTPase-null virus (Figure 7C, 7D). However, this deficit is largely rescued in IFNAR−/− mice (Figure 7E), supporting the conclusion that it is the anti-IFN but not the dUTPase activity of ORF54 that plays a major role for MHV-68 establishment of latent infection of mice.

Across eukaryotes, prokaryotes, and viruses, dUTPases are highly conserved proteins, especially at the structural level [56]. Most eukaryotic dUTPases have 5 conserved motifs ordered 1–5 from the N- to C- terminal [45], [48], [49]. Functional dUTPases are formed as homotrimers containing three complete active sites formed from motifs 1–5 of each protein [45], [49]. However, the herpesvirus monomer is an active dUTPase with one active site formed by motifs 1–5 [48], [57]. The herpesvirus dUTPase protein sequence is twice as large as the cellular, with the C-terminal half containing domains 1, 2, 4, and 5 [48] and a herpesvirus unique domain called motif 6 between motifs 2 and 4. Towards the center of the protein, there is an actual motif 3, that was maintained after the loss of motifs 1, 2, 4, and 5 from the N-terminal portion [45], [48]. By examining the ability of murine cellular dUTPase to inhibit type I IFN signaling in our reporter assay, we found that dUTPase activity alone does not diminish IFN-α induced activation of ISRE, while an ORF54 dUTPase-null mutant still does (Figure 1). Therefore, it is possible that the N-terminal half of ORF54 may have evolved the additional function of type I IFN inhibition. For the gammaherpesviruses MHV-68, KSHV, and EBV, the ORF54 motif 3 starts at amino acid 82, 78, and 73, respectively. Since the motifs required for active site formation are found by the C-terminal halves of each protein, the N-terminal portion upstream of motif 3 in herpesviral dUTPases contain no recognizable or conserved sequences from cellular dUTPases. The sequences in the N-terminal half of ORF54 demonstrate some conservation between the gammaherpesviruses examined, but further studies designed to functionally map ORF54 anti-IFN activity are required to identify such a domain. ORF54 is an early protein [58] that, like other viral dUTPases, is expressed in both the cytoplasm and nucleus of infected cells, while cellular dUTPases are only found in the nucleus and mitochondria of cells [59]. Using a recombinant MHV-68 with a FLAG epitope tag on ORF54 to study its expression kinetics, we found that during early stages of infection ORF54 appears evenly distributed, but has preference for the nucleus at late stages of virus replication when cytopathic effect is obvious (Figure S3). As the MHV-68 genome is replicated in the nucleus, perhaps ORF54 functions as a dUTPase in the nucleus to help maintain genomic integrity and as an inhibitor of type I IFN signaling in the cytoplasm. It would be informative to identify ORF54 protein interaction partners in both compartments and at the various stages of virus infection.

Interestingly, beta- and gammaherpesviruses contain several ORFs with dUTPase-related domains that demonstrate strong divergence, where besides their catalytic motifs they exhibit different functions [48], some involving innate immune responses. Betaherpesviruses are unique in that many do not have a single ORF with dUTPase catalytic activity [60], [61]. However, several betaherpesvirus ORFs exist with dUTPase-related domains, such as UL72, UL82, UL83, UL84, and UL31 [48]. Human cytomegalovirus (HCMV) encodes pp65, also called UL83, that contains the dUTPase-related domain motif 6 [48]. HCMV pp65 expression led to the inhibition of phosphorylation of IRF3 and to its sequestration in the cytoplasm following induction of the IFN pathway [62], [63]. In gammaherpesviruses, although ORF54 is the only functional dUTPase, ORFs 10 and 11 both contain the dUTPase-related domain motif 6 [45], [48]. Here we showed in a transient transfection reporter assay that KSHV ORF54 is capable of reducing IFN-α responses (Figure 1). However, EBV ORF54 was found to induce expression of several pro- and anti-inflammatory cytokines when cells were treated with purified EBV ORF54 [64]–[67]. The biological significance of immune modulation by EBV ORF54 in the context of virus infection remains to be determined. KSHV ORF10 is a viral lytic protein that blocks IFN signaling by forming inhibitory complexes with JAK1 and TYK2 [35]. EBV ORF11 (LF2) was found to bind to IRF7 to inhibit dimerization and IRF7-mediated activation of type I IFN production, in a manner unrelated to its dUTPase domain [54]. Previously, we also found that MHV-68 ORF11 has a similar effect to ORF54 in inhibiting ISRE reporter induction by IFN-α [34]. Therefore, it is possible for large DNA viruses, such as herpesviruses, to utilize multiple proteins to inhibit type I interferon responses. This is further supported by the observation that STAT1 activation upon IFN-α treatment is not fully recovered in cells infected with the 54Stop virus (Figure 4B), indicating the presence of other viral proteins with overlapping anti-IFN functions.

Our results have revealed a previously unrecognized and critical anti-IFN function of ORF54, but they also raise a question about the biological role of its dUTPase activity. Numerous RNA and DNA viruses, such as retroviruses, poxviruses, and herpesviruses, encode a functional dUTPase in their genomes, suggesting its importance for the viral life cycle [45], [68], [69]. However, viral dUTPases are generally dispensable for virus replication [50], [70], [71], likely because cellular dUTPase is readily available and active in most dividing cells. Many cellular enzymes involved in DNA replication and nucleotide metabolism are strongly cell-cycle dependent. Therefore, it is presumably advantageous for the virus to encode its own enzymes for genome replication in terminally differentiated and non-dividing cells where the cellular counterparts may not be available. For example, herpesviruses encode several other enzymes in addition to dUTPase, such as thymidine kinase (TK), exonuclease, Uracil-DNA glycosylase (UNG), and ribonucleotide reductase (RR). For MHV-68, these viral enzymes are not required for *in vitro* virus replication in actively dividing cultured cells, but disruption of TK or RR leads to severe attenuation in acute productive infection in the lung as well as in splenic latency [50], [72], [73]. However, because site-specific mutations targeting the enzymatic activity of these proteins were not used in these studies, it cannot be completely excluded that other possible mechanisms unrelated to their known TK or RR functions account for the observed severe attenuation.

In this study, we constructed a dUTPase-null virus, 54DM, to study the specific contribution of the enzyme activity of ORF54. Interestingly, unlike with TK and RR, lack of dUTPase activity or even the entire ORF54 protein does not detrimentally retard virus replication in the lungs of infected mice. Previous studies in our lab have found that ORF54-null viruses constructed with a large transposon insertion cause a more significant defect on lytic replication in the lungs of infected mice [50]. This discrepancy may be due to the large disturbance each transposon causes in the highly compact MHV-68 viral genome, where most promoter regions overlap coding regions. The ORF54-null virus, 54Stop, shows a very strong defect in the infectious center assay that is not found with the dUTPase-null virus, implying that the lack of ORF54 enzymatic activity does not dramatically hinder the establishment of the MHV-68 latent load, while lack of the entire protein does. However, mice infected with 54DM do have a slight, but statistically insignificant, reduction in the amount of reactivated virus from infected splenocytes, at 2.8- and 2.4-fold defect compared to WT and 54R viruses, respectively (Figure 7C). This marginal defect of 54DM was not observed when the viral genome copy number was analyzed (Figure 7D), thus it is possible that the phenotype seen in the infectious center assay is due to the lack of dUTPase during reactivation. The 54Stop virus lacks any expression of ORF54, including its dUTPase activity, and the 2.8-fold reduced level of infectious centers compared to 54R in IFNAR−/− mice mirrors that seen with 54DM virus during reactivation in Balb/C mice. Indeed, as with 54DM in wild-type mice, 54Stop has nearly identical viral genome copies as 54R in the splenocytes of IFNAR−/− mice, again suggesting that the minor defect in the infectious center assay is due to reactivation. Although it was expected that dUTPase-null viruses grow normally in actively dividing cell culture, it is surprising that the only phenotype we observed in mouse infection is a moderate defect in reactivation from latency. However, our observation is not unique. Following *in vivo* infection, HSV-1 dUTPase mutants replicate like wild type in the footpad, sciatic nerve, and dorsal root ganglia; a defect of about 10- to 100-fold is only visible when the virus moves to the CNS spinal cord. These dUTPase mutants were able to establish latent infections but demonstrated a defect in reactivation from latency [74]. Taken together, our results indicate that the dUTPase activity of ORF54 does not have a significant role during MHV-68 productive infection in the lung or latency in the spleen. However, our interpretation for the maintenance of dUTPase in the viral genome is that dUTPase activity is likely required for genome stability over many generations. Furthermore, reactivation is considered much less effective than lytic replication in permissive cells, thus a less optimal replication environment may have a more profound effect on the reactivation process over time.

Gammaherpesviruses are characterized by their ability to establish latent infection in lymphocytes. Establishment of viral latency first requires the efficient infection of lymphocytes. By affecting persistent infection of gammaherpesviruses, the IFN response has far reaching effects on the viral life cycle and the establishment of latency, instead of only acting primarily on initial infection. Furthermore, the type I IFN response is critical in shaping the adaptive immune response [1], [2], [6], [9], [36]. Altering the balance between the host immune response and viral immune evasion genes could drastically affect the overall outcome of viral infection. The anti-IFN function of ORF54 appears to be relatively dispensable for lytic replication *in vitro* and *in vivo*, but absolutely required for the establishment of latency. As a whole, our data suggests that evading the type I IFN response is critical for the establishment of latent infection in lymphocytes and that ORF54 plays a major role in this evasion. The reduced latency observed in the infection of 54Stop may be due to the inability of the virus to replicate well in a particular cell type, such as lymphocytes, that is more sensitive or responsive to the anti-viral effects of IFN. Although several genes are likely required and may have some overlapping functions, lack of one of these genes, such as ORF54, retards establishment of latent infection. Our results in IFNAR−/− mice not only emphasize the importance of ORF54 in the type I IFN pathway by demonstrating a rescue in an infectious center assay, but also hint at the necessity of this host pathway in blocking the establishment of latent infection in lymphocytes. However, the mechanisms by which the virus spreads from the inoculation and lytic replication sites to the latency compartment remain largely elusive. Thus, although our data suggests its importance, we are currently unable to isolate at which stage during this viral spread the anti-IFN function of ORF54 is required. Analysis with a detailed time course and different cell types is required to further understand the interaction between type I interferon responses and latency establishment.

Defining immune evasion strategies employed by MHV-68 will allow better design of a live attenuated vaccine to human gammaherpesviruses KSHV and EBV. One strategy to increase the success of a vaccine is to limit the establishment of latency and increase its immunogenicity by removal of viral immune evasion genes [75]. Because ORF54 is not required for virus replication, plays a role in type I IFN inhibition, and is necessary for the efficient establishment of latency, a virus lacking ORF54, as well as other immune evasion genes and genes required for latency, is a promising vaccination strategy.

## Materials and Methods

### Ethics statement

This study was carried out in strict accordance with the recommendations in the Guide for the Care and Use of Laboratory Animals of the Public Health Service (National Institutes of Health). The protocol was approved by the Institutional Review Board and Animal Research Committee of the University of California Los Angeles (assurance of compliance number: A3196-01). All procedures were performed under ketamine and xylazine anesthesia and all efforts were made to minimize suffering.

### Cells

293T and Vero cells were cultured in complete Dulbecco's modified Eagle medium (DMEM) containing 10% FBS. NIH3T3 cells were cultured in DMEM containing 10% BCS. Bone-marrow derived macrophages (BMDM) from both wild-type and IFNAR−/− mice were immortalized by v-raf and c-myc oncogenes, cultured in RPMI containing 10 mM HEPES, 10% FBS, and MCSF, and were a kind gift from Shankar Iyer (Genhong Cheng Lab, UCLA). All culture medium was also supplemented with penicillin and streptomycin and all cells were cultured at 37°C with 5% CO_2_.

### dUTPase assay for enzymatic activity

dUTPase assay was adapted and modified from previous methods used to measure enzymatic activity [44], [59], [76]. Briefly, each potential FLAG-tagged dUTPase construct was transfected into 293T cells using Lipofectamine 2000 (Invitrogen). 48 hours post transfection, cells were lysed with buffer containing 100 mM Tris pH 7.5, 50 mM NaCl, and 1% NP-40, supplemented with 1 mM PMSF, 1 µg/mL Aprotinin, 1 µg/mL Leupeptin, 1 µg/mL Pepstatin A, 1 mM Na_3_VO_4_, and 1 mM NaF. Lysates were kept on ice for 10 minutes and clarified with a high speed spin at 4°C for 15 minutes. FLAG-tagged proteins were immunoprecipitated by incubating lysates with Protein G Sepharose (GE Healthcare 17061801) and 4 µg of anti-FLAG antibody (Sigma F3165), followed by elution from the antibody by incubation with 20 µg of 3×FLAG peptide (Sigma F4799). 5 ul of purified FLAG-tagged proteins were incubated at 37°C with 5 ul of 5 mM dUTP (Promega U1191) for 0, 2, 5, 10, 15, 20, and 25 hours in 10 ul of 2× reaction buffer comprised of 100 mM Tris pH 7.5, 20 mM MgCl_2_, 20 mM DTT, and 0.2 mg/mL BSA. Reactions were terminated by freezing. Potentially digested dUTP are at a maximum final concentration of 1.25 mM, lower if digested. PCR was conducted using MHV-68 BAC DNA as a template, with primers to amplify a region in ORF57 (forward primer: 5′-ACTGAAACCTCGCAGAGGTCC-3′ and reverse primer 5′-GCACGGTGCAATGTGTCACAG-3′) using the potentially digested dUTP alongside dATP, dCTP, and dGTP. Cycle conditions were 95°C 5 min; 95°C 30 seconds, 60°C 30 seconds, 72°C 30 seconds for 35 cycles; 72°C 10 minutes; hold at 4°C. PCR products were run on a 3% agarose gel.

### Generation of recombinant MHV-68

The recA^+^ *Escherichia coli* (*E. coli*) strain GS500 harboring a BAC containing the wild-type MHV-68 genome was used to construct recombinant MHV-68 by allelic exchange with the conjugation competent *E. coli* strain GS111 containing a suicide shuttle plasmid pGS284, as previously described [34], [77], [78]. For each recombinant MHV-68, overlap extension PCR was used to construct a unique shuttle plasmid pGS284 harboring the desired mutation with a 500 base pair flanking region. To screen for the correct mutation, restriction enzyme digests were performed on PCR products amplified with the outer primer sets on the BAC MHV-68 clones. WT MHV-68 and 54R contain a *BamH*I restriction enzyme site that is altered to a *Hind*III site in 54Stop. The base pair changes in 54DM allow the introduction of an *Ase*I site that is absent in WT MHV-68. Following selection of the desired recombinant clone, the MHV-68 BAC DNA was purified and transiently transfected with Lipofectamine 2000 into 293T cells with an equal amount of a plasmid expressing Cre recombinase to remove the BAC sequence. Three days post transfection, a single viral clone was isolated by limiting dilution and propagated for future studies. Produced viruses were quantified by plaque assay and limiting dilution. Genomic integrity of the final recombinant viruses was analyzed by *Sma*I and *EcoR*I restriction enzyme digestion and Southern blot with DIG-labeled DNA probes to the whole genome or 6 kb of the left end (Roche 11585614910). Primers used for the construction of each shuttle plasmid are listed 5′ to 3′ as follows: 54R and 54Stop, outer primers: CAGGACAGATCTCACTAGACACTGTGACTATAGAC and CTGTCCCCCGGGTAGCAGACACAGGTCCTCAG; 54Stop, inner primers: CACATAACTGAGTAAGCTTACCAGACTAAAATTTCAAAACATCAC and TGGTAAGCTTACTCAGTTATGTGCCTTGTGTAGTTAGGGCC; 54DM, outer primers: GAAGAGATCTCACTCCCCCACTGAGGGACGTATGTGTCAGC and GTTGGCTAGCCAATTTCAGACTTGTCTGCAGCTTCGTGCGGAACCCTAATAAAC; 54DM, inner primers: ATTAATCAGTCCAGTTGCGCCGGTGACGAGACTGTT and GCAACTGGACTGATTAATCCCGGCTACCGGGGTGAAAT.

### MHV-68 virus quantification

MHV-68 concentrated virus was titered using plaque assay and limiting dilution. For plaque assay, 10-fold serial dilutions of each virus were incubated on a monolayer of Vero cells for 1 hour. The infected cells were overlaid with 5% methylcellulose DMEM. At 6 days post infection, cells were fixed with 2% crystal violet in 20% ethanol. Plaques were counted at the optimum dilution to calculate virus titer. To determine the 50% Tissue Culture Infectious Dose (TCID_50_), 12 wells seeded with Vero cells in a 96-well plate were infected by 100 ul of each 10-fold serial dilution. 7 to 10 days post infection, every well was scored for cytopathic effect (CPE). TCID_50_ was determined by the calculation TCID_50_/mL = 10×10∧[(highest dilution with 100% CPE)−[0.5+((# of wells with CPE/total wells) in next highest dilution)+(each following dilution's CPE fraction)]]. Viral titers of multiple step growth curves were quantified by plaque assay.

### Plasmids

Wild-type MHV-68 BAC was constructed as previously described [34]. The firefly luciferase reporter driven by the interferon-stimulated response element (ISRE_firefly-luciferase) and the renilla luciferase reporter driven by the promoter of housekeeping gene phosphoglycerate kinase (PGK_renilla-luciferase) were kind gifts from Dr. Genhong Cheng and Dr. Lily Wu, respectively (UCLA). All MHV-68 open reading frames were cloned into pENTR (Invitrogen) by PCR amplification from MHV-68 DNA, then transferred to a modified destination vector resulting in a 3×FLAG epitope tag at the N-terminus. Specifically, for MHV-68 ORF54, primers used are 5′-CCGAGCGAATTCAATGAAAGTGGAATACTCCTTTGTG-3′ and 5′-GCTCGGGGTACCTTAATTCACCCCACTTGACCCAAAC-3′. To construct ORF54 MHV-68 H80A/D85N by overlap extension PCR, the same inner primers as for 54DM virus were used, the outer primers used are 5′-CCGAGCGAATTCAATGAAAGTGGAATACTCCTTTGTG-3′ and 5′- GCTCGGGGTACCATTCACCCCACTTGACCCAAAC -3′. Wild-type ORF54 and ORF54 H80A/D85N have slightly different molecular weights due to the cloning process of FLAG-ORF54 H80A/D85N. A modified Gateway cloning system (Invitrogen) was utilized to generate entry clones of the target protein, then recombined by an LR reaction into the same destination vector as FLAG-ORF54. This process inserted 6 additional amino acids to the N-terminus of the protein (from the multiple cloning site) after the 3×FLAG tag and before the ORF54 H80A/D85N ATG start site. Also, the H80A/D85N clone utilizes a STOP codon in the destination vector while the FLAG-ORF54 clone includes the endogenous STOP codon. Therefore, 10 additional amino acids (from the multiple cloning sites) are added to the C-terminus of FLAG-ORF54 H80A/D85N. In total, the FLAG-ORF54 H80A/D85N protein has an additional 16 amino acids, or approximately 1.7 kDa additional molecular weight, compared to the wild type FLAG-ORF54.These changes demonstrated no effect on ORF54 or ORF54 H80A/D85N function. KSHV ORF54 was cloned from the a KSHV BAC construct, a kind gift from Dr. Jae Jung (University of Southern California). The primers used are 5′-TAATGGATCCATGAACAACCGCCGAGGCTC-3′ and 5′-TAATGTCGACCTAAAACCCAGACGACCCCAG-3′.

### Promoter reporter assay

293T cells were seeded at 5×10^4^ cells per well in a 48-well plate 16 hours prior to transfection. Cells in each well were transfected using Lipofectamine 2000 (Invitrogen), with 20 ng of ISRE_firefly-luciferase, 5 ng of PGK_renilla-luciferase, 200 ng of viral ORF or vector control, and 175 ng of filler DNA. 24 hours post transfection, identically transfected wells were left untreated or were treated with 3×10^4^ units of human interferon-α (both human and mouse IFN-α are purchased from pbl Interferon Source). 24 hours post treatment, both firefly and renilla luciferase activity was measured (Promega Dual-Luciferase Assay Kit). Fold activation was calculated by first normalizing all values to their internal renilla luciferase control, and then by dividing luciferase activity in the treated samples by that of the untreated samples. Expression of viral ORFs was determined by immunoblot against the FLAG epitope expressed at the N-terminus of each of the ORFs in our MHV-68 expression library.

### Immunofluorescence assay

NIH3T3 cells were seeded in 24-well plates and infected with FLAG-54 MHV-68 at an MOI of 1. Cells were fixed and permeabilized with treatment for 30 minutes at room temperature with 100% methanol. Cells were then washed in three changes of PBS and stored in PBS until staining. Mouse anti-FLAG M2 (Sigma F3165) was incubated with cells overnight for 16 hours at a dilution of 1∶750, and Alexa Fluor 594 goat-anti mouse IgG (Invitrogen A11005) at a dilution of 1∶1000 was incubated with cells for 1 hour. Hoechst dye was added for 10 minutes prior to analysis.

### Immunoblot

Cells were lysed for 10 minutes on ice in lysis buffer (50 mM Tris pH 7.5, 1% NP-40, 0.25% sodium deoxycholate, 150 mM NaCl, 1 mM EDTA) supplemented with 1 mM PMSF, 1 mM Na_3_VO_4_, and 1 mM NaF. Lysates were then combined with 4× protein sample buffer (0.25 M Tris pH 6.8, 8% SDS, 40% glycerin, 20% β-mercaptoethanol, 0.008% Bromophenol blue), sonicated, and boiled for 10 minutes prior to loading on a 10% polyacrylamide gel. Membranes were stripped with Multi-Western Stripping buffer (Bioland Scientific), prior to re-probing with each subsequent antibody. The antibodies used in this study were rabbit anti-human phosphoSTAT1 (Cell Signaling 9167), rabbit anti-mouse phosphoSTAT1 (Millipore 07307), rabbit anti-total STAT1 (Cell Signaling, 9175S), mouse anti-FLAG M2 (Sigma F3165), rabbit anti-human IFNAR1 (Abcam, ab45172), mouse anti-β-actin (Sigma A5316), rabbit anti-IGF-1 Receptor β (Cell Signaling 3027S), rabbit anti-IFNAR2 (Novus Biologicals 31665), rabbit anti-phosphoErk1/2 (Cell Signaling 4376), and rabbit anti-IFIT2 (Abcam 55837). Rabbit serum against viral lytic protein ORF65 was derived in our lab. Secondary antibodies conjugated to HRP were donkey anti-rabbit IgG (GE Healthcare NA934V) and sheep anti-mouse IgG (GE Healthcare NXA931).

### Real-time PCR

Total RNA was extracted from cells by RNeasy Mini Kit (Qiagen) and reverse transcribed into cDNA by qScript cDNA Synthesis Kit (Quantas). Primers used in RT-PCR to quantify cellular transcripts are as follows: actin: 5′-GTATCCTGACCCTGAAGTACC-3′ and 5′-TGAAGGTCTCAAACATGATCT-3′; human IFNAR1: 5′- AACAGGAGCGATGAGTCTGTC-3′ and 5′- TGCGAAATGGTGTAAATGAGTCA-3′; murine IFNAR1: 5′- AGACGAGGCGAAGTGGTTAAA-3′ and 5′- GCTCTGACACGAAACTGTGTTTT-3′; murine MX1: 5′-GAATAATCTGTGCAGGCACTATGA-3′ and 5′-CTCTCCACTCCTCTCCTTCTTTC-3′; murine IFIT1: 5′-TGCTTTGCGAAGGCTCTGAAA-3′ and 5′-TTCTGGATTTAACCGGACAGC-3′; murine IFIT3: 5′-AGTGAGGTCAACCGGGAATCT-3′ and 5′-TCTAGGTGCTTTATGTAGGCCA-3′.

### *In vivo* assays

All *in vivo* procedures were performed according to protocols approved by the University of California, Los Angeles, the Animal Research Committee, and the Institutional Review Board. Balb/C mice were purchased from Charles River Laboratories. IFNAR−/− mice were a kind gift from Dr. Genhong Cheng at UCLA. Mice were intranasally infected with 500 pfu under sedation by I.P. injection of 2 mg ketamine and 0.04 mg xylazine. At 5 and 7 days post infection, mice were sacrificed and lung tissue was harvested in 1 mL of complete DMEM. Lung tissue was homogenized to measure the viral titer by plaque assay. At 14 days post infection, mice were sacrificed for infectious center assay of splenocytes. Briefly, a single cell suspension was isolated from the spleen of each infected animal. The splenocytes were co-cultured for one day with a monolayer of Vero cells, then overlaid with 5% methylcellulose DMEM for 6 additional days prior to fixing cells with 2% crystal violet in 20% ethanol. Each viral plaque reflects MHV-68 reactivated from splenocytes. Plaques were counted at the optimum dilution and number of infectious centers calculated per 1×10^7^ splenocytes. To determine viral genome copies, total genomic DNA for quantitative-PCR was harvested from lung lysates and splenocytes using QiaAmp DNA Mini Kit (Qiagen). The PCR reaction was comprised of 100 ng of total genomic DNA as a template and the primers used were 5′-ACCTTGAAACCCGTGAAGG-3′ and 5′-CATCTGCCACGACCTGAGAT-3′.

### Gene ID numbers

Swiss-Prot accession numbers for the proteins/genes used in this study are as follows: MHV-68 ORF54, P88991; KSHV ORF54, P88942; MHV-68 ORF48, P88986; and murine cellular dUTPase (DUT), Q9CQ43. The MHV-68 viral genome GenBank accession number is U97553.2.

## Supporting Information

Figure S1 **ORF54 does not alter the transcript level of IFNAR1 during infection.** 293T and NIH3T3 were infected at MOI 1 for 24 hours, and bone marrow derived macrophages from wild type mice (BMDM) were infected at MOI 2 for 24 hours. Equal infection was ensured by RT-PCR quantifying input viral genome copies from total infected cellular DNA 1 hour after virus was introduced to the culture. RNA was harvested and reverse transcribed to cDNA. Human (293T) and murine (NIH3T3, BMDM) IFNAR1 transcript levels were quantified by RT-PCR, normalized first to actin, and are shown relative to uninfected cells. Similar to P-values of 54Stop, 54DM, and 54R compared to WT infection are not significant for any of the three cell types. UI = uninfected.
(TIF)

Figure S2 **Viral genome copies isolated from infected lung lysates.** Balb/C mice were infected with 500 pfu of each indicated virus. Lung tissue was harvested for isolation of DNA to measure viral genome copy number by quantitative RT-PCR at **A**) 5 dpi and **B**) 7 dpi. P-values for 54S compared to WT, 54DM, and 54R in A are 0.025, 0.013, 0.003, respectively and in B are 0.561, 0.052, 0.190, respectively.
(TIF)

Figure S3 **Expression kinetics and localization of ORF54 during infection.** NIH3T3 cells were infected at MOI 1 with an MHV-68 recombinant virus with an N-terminal FLAG tag on ORF54. **A**) Immunoblots against FLAG epitope demonstrate ORF54-FLAG expression. Immunoblot was stripped and re-probbed for β-actin as a control. **B**) Immunofluorescence assay demonstrating the localization of ORF54-FLAG during infection. Hoechst was used to stain nuclei. UI = uninfected cells, hpi = hours post infection.
(TIF)
