# Supplementary figures and images for: The Anti-interferon Activity of Conserved Viral dUTPase ORF54 is Essential for an Effective MHV-68 Infection
Source: PLoS Pathog. 2011 Oct 6;7(10):e1002292. doi: 10.1371/journal.ppat.1002292 (PMC3188543; doi:10.1371/journal.ppat.1002292)

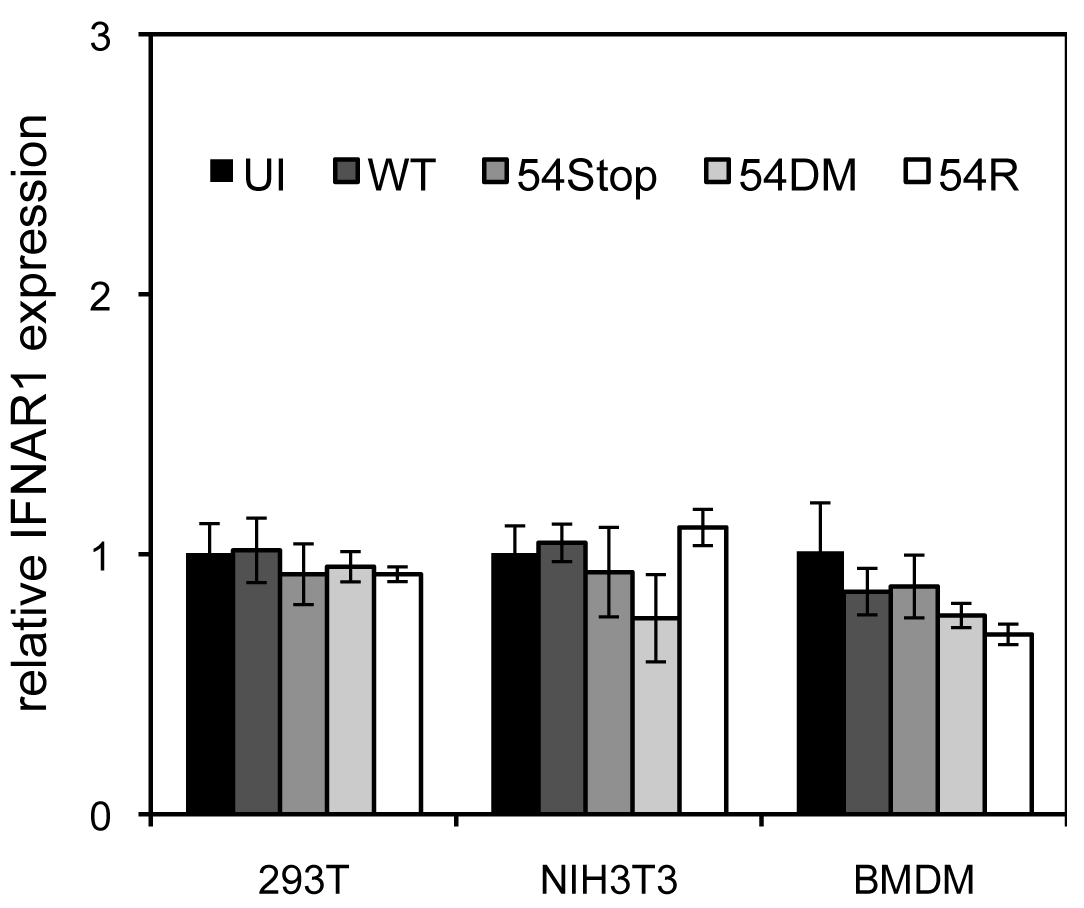

Supplement: Figure S1 — ORF54 does not alter the transcript level of IFNAR1 during infection. 293T and NIH3T3 were infected at MOI 1 for 24 hours, and bone marrow derived macrophages from wild type mice (BMDM) were infected at MOI 2 for 24 hours. Equal infection was ensured by RT-PCR quantifying input viral genome copies from total infected cellular DNA 1 hour after virus was introduced to the culture. RNA was harvested and reverse transcribed to cDNA. Human (293T) and murine (NIH3T3, BMDM) IFNAR1 transcript levels were quantified by RT-PCR, normalized first to actin, and are shown relative to uninfected cells. Similar to P-values of 54Stop, 54DM, and 54R compared to WT infection are not significant for any of the three cell types. UI = uninfected. (TIF) [file ppat.1002292.s001.tif]

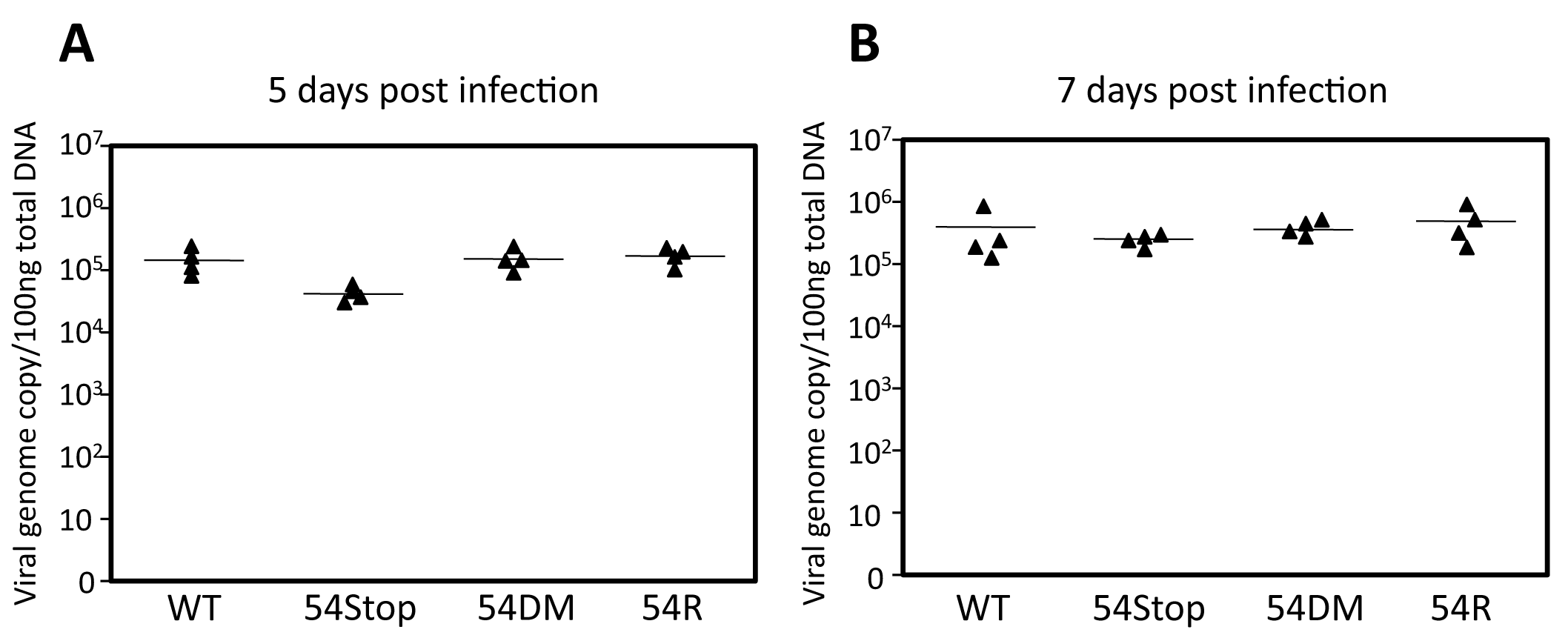

Supplement: Figure S2 — Viral genome copies isolated from infected lung lysates. Balb/C mice were infected with 500 pfu of each indicated virus. Lung tissue was harvested for isolation of DNA to measure viral genome copy number by quantitative RT-PCR at A) 5 dpi and B) 7 dpi. P-values for 54S compared to WT, 54DM, and 54R in A are 0.025, 0.013, 0.003, respectively and in B are 0.561, 0.052, 0.190, respectively. (TIF) [file ppat.1002292.s002.tif]

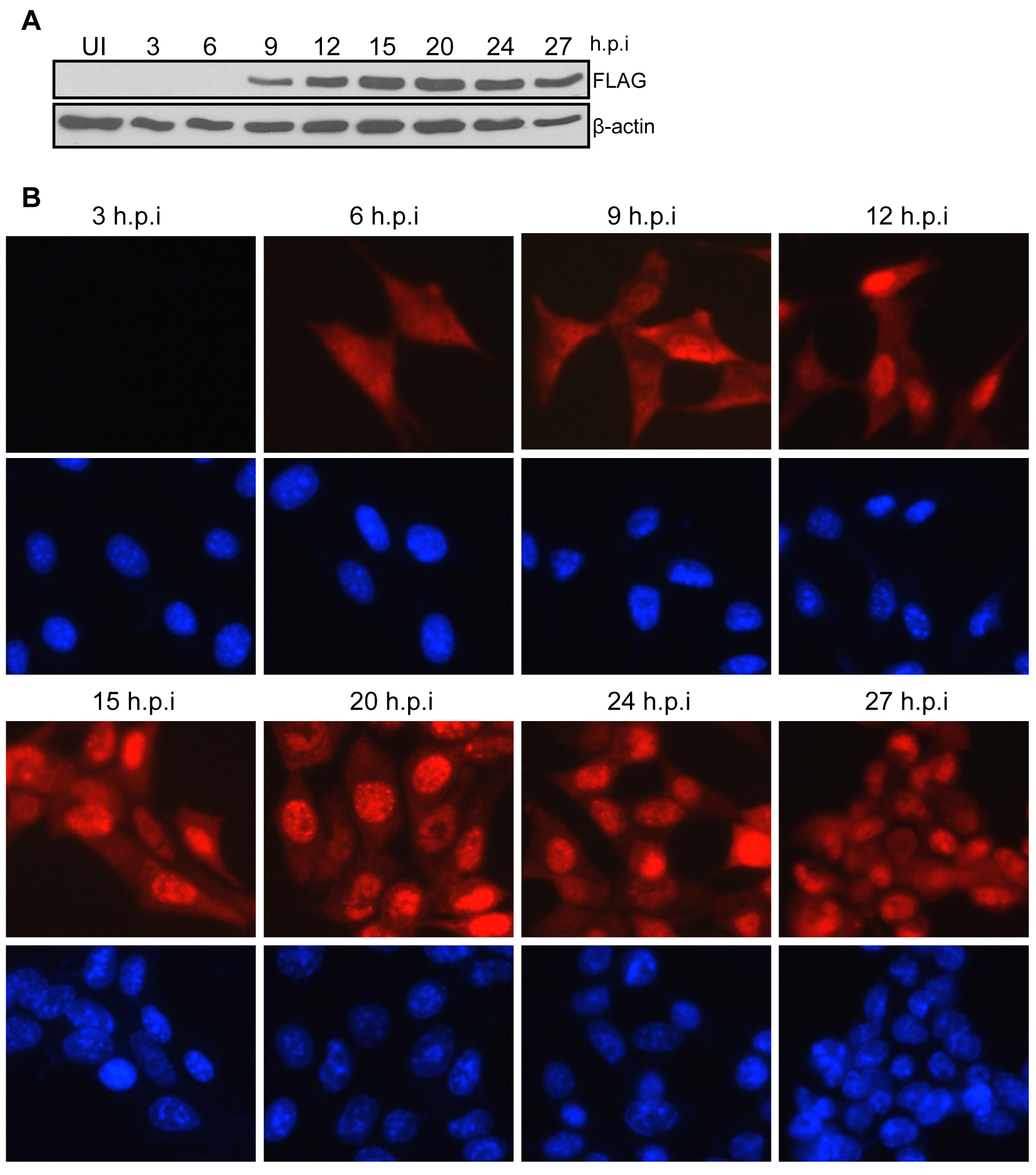

Supplement: Figure S3 — Expression kinetics and localization of ORF54 during infection. NIH3T3 cells were infected at MOI 1 with an MHV-68 recombinant virus with an N-terminal FLAG tag on ORF54. A) Immunoblots against FLAG epitope demonstrate ORF54-FLAG expression. Immunoblot was stripped and re-probbed for β-actin as a control. B) Immunofluorescence assay demonstrating the localization of ORF54-FLAG during infection. Hoechst was used to stain nuclei. UI = uninfected cells, hpi = hours post infection. (TIF) [file ppat.1002292.s003.tif]
